# “Second victims” in Covid-19 pandemic: A cross-sectional study among medical doctors of the Catanzaro Hospital

**DOI:** 10.1192/j.eurpsy.2022.1345

**Published:** 2022-09-01

**Authors:** A. Zibetti, C. Scalise, M.A. Sacco, P. Ricci, I. Aquila

**Affiliations:** Institute of Legal Medicine, Medical And Surgical Science, Catanzaro, Italy

**Keywords:** medical malpractice, medical errors, second victim, defensive medicine

## Abstract

**Introduction:**

Medical errors are a serious public health problem. The COVID-19 pandemic has caused further stress to doctors with the increase in patient mortality, the lack of definite guideline and growing work demands. In this scenario, the patient is not the only victim of the medical error. The “second victim” (SV) is defined as a health worker who was involved in an unforeseen and negative event for the patient, who suffers physically and psychologically, because he was traumatized by his own mistake and/or by the injuries caused to the patient. The SV phenomenon prevalence varies from 10.4% up to 43.3%.

**Objectives:**

The aim of this study is to evaluate the second victim phenomenon during the COVID-19 pandemic among medical doctors of the Catanzaro University Hospital (Italy).

**Methods:**

A cross-sectional study will be conducted by administering an anonymous questionnaire to the Catanzaro University Hospital medical doctors using SurveyMonkey software. Descriptive analysis will be performed.

**Results:**

The data collection is ongoing. Currently, 300 subjects are included in the sample.

**Conclusions:**

The second victim phenomenon has a negative impact on doctors, colleagues and patients. It is important to aid health workers involved in an adverse event by activating support networks and adopting appropriate strategies in order that the event is a source of learning and not of demotivation.

**Disclosure:**

No significant relationships.

